# Preface to the theme issue ‘Uncertainty quantification for healthcare and biological systems (Part 2)’

**DOI:** 10.1098/rsta.2024.0231

**Published:** 2025-04-02

**Authors:** Louise M. Kimpton, L. Mihaela Paun, Mitchel J. Colebank, Victoria Volodina

**Affiliations:** ^1^ Department of Mathematics and Statistics, University of Exeter, Exeter, UK; ^2^ School of Mathematics and Statistics, University of Glasgow, Glasgow, UK; ^3^ School of Mathematical Sciences, University of Southampton, Southampton, UK; ^4^ Edwards Lifesciences Foundation Cardiovascular Innovation and Research Center, Department of Biomedical Engineering, University of California, Irvine, CA, USA; ^5^ Department of Mathematics, University of South Carolina, Columbia, SC, USA

**Keywords:** uncertainty quantification, biology and healthcare, clinical decision support, mechanistic models, digital twins

Biological and healthcare system models are beginning to be used for decision support in the form of medical simulations and digital twins. These simulations and digital twins can explain the driving mechanisms behind physiological processes and predict outcomes thus having the potential to revolutionize personalized healthcare. However, when these models are used to make patient- or population-specific statements, it is crucial to quantify the different sources of uncertainty in the system to enable reliable model-based inference and clinical decision support. Our special issue aims to demonstrate, on biological systems and healthcare real-world applications, how to identify and account for uncertainties in a model-based analysis. Our special issue strives to help researchers in the healthcare/biological modelling field understand the significance of uncertainty quantification (UQ) and encourage the adoption of UQ as part of the credibility assessment, as recommended by regulatory agencies in the US and EU.

Here we provide an introduction to the theme issue ‘Uncertainty quantification for healthcare and biological systems (Part 2)’. The articles in volume 2 of our special issue continue to address multiple challenges in the application of UQ to biological and healthcare models raised in our review article on ‘Challenges and opportunities in uncertainty quantification for healthcare and biological systems’ [1], which is the introduction to volume 1 of our theme issue.

The review article by Goldstein *et al*. [2] provides theoretical and practical implementation details of model discrepancy, which is an often neglected source of uncertainty in the field of healthcare and biological modelling. The article by Grigorian *et al*. [3] expands on this by proposing a hybrid ‘grey-box’ approach to model discrepancy. The authors combine a clinical model of the oxygen dissociation curve with a neural network model to learn missing biophysical processes and compare their approach with standard black-box type methods based on Gaussian Processes.

The study by Schmid *et al*. [4] considers scientific machine learning in the context of universal differential equations (UDEs). The authors investigate several epistemic UQ methods for the joint inference from data of parameters from a mechanistic model and neural network component of a UDE. In the field of emulation, the study by Kimpton *et al*. [5] devises a new sequential design strategy for a stochastic COVID-19 model as an alternative to existing methods that select new design points through optimization rather than a grid search. They show that the new approach outperforms current methods when the stochastic noise is large.

To address the critical need for new UQ methods for spatial agent-based models, Gamal *et al*. [6] propose a spatio-temporal uncertainty propagation technique for agent-based models. The method computes ensemble and observational uncertainties via a propagation Sobol index, which are applied to a population model of tobacco purchase.

Applebaum *et al*. [7] aim to assist clinicians in forecasting the progression of the Duchenne Muscular Dystrophy disease by developing a new model for predicting North Star Ambulatory Assessment (NSAA) scores, along with measures such as the 10 m walk time and the time taken to rise from the floor for patients suffering from this disease. The authors propose a dynamic linear model to predict the trajectories of these clinical outcomes. In addition, the authors evaluate the effectiveness of the proposed models in generating synthetic NSAA score datasets.

On the topic of parameter identifiability, Foster & Ellwein Fix [8] study parameter identification in a nonlinear respiratory mechanics model specific to preterm infants. The respiratory model predicts pulmonary volumes, pressures and airflows under varying levels of continuous positive airway pressure. The parameter identification methodology uses both global sensitivity analysis (via Morris’ screening) and local sensitivity analysis combined with a singular value decomposition-based subset selection. The strategy identifies key model parameters affecting specific outputs and fits them to experimental pulmonary data from healthy, preterm infants using gradient-based optimization. This method produces patient-specific parameters that align model predictions with the data, demonstrating its feasibility. In addition, the article by Tong *et al*. [9] presents a neural-network based, data-driven framework for enhanced digital twin analysis. The authors develop and apply a framework for detecting non-identifiable manifolds for parameter inference, addressing the critical issue of parameter identifiability within the context of a cardiovascular model.

A further article on parameter identifiability is that by Hilhorst *et al*. [10], which focuses on increasing the awareness of the effectiveness of *in silico* clinical trials. To do this, they apply sensitivity analysis (SA) to understand the input–output relationships of a virtual patient cohort (VPC) created using a virtual cohort generator (VCG). The VCG is able to simulate outputs that mimic the physiological responses of real patients while ensuring that the outputs are physiologically plausible and represent a diverse set of patients. In the article, they use a one-dimensional pulse wave propagation model of the coronary circulation, developed as part of a VCG with synthetic coronary artery disease patients. Applying SA explores input–output relationships as well as providing validation for the VPC. Additionally, the study by Hiremath *et al*. [11] assesses the accuracy and identifiability of parameters in a mathematical model of high-grade glioma. The authors use virtual patient cohorts based on a family of biology-based mechanistic models and use a model-selection framework to identify models most consistent with their clinical data. The authors show that under noisy conditions, they are able to identify 11 out of the 12 total parameters in their system.

In the field of epidemiology, Ogi-Gittins *et al*. [12] propose a simulation-based approach for estimating reproduction number ( $R_{t}$ ) during infectious disease outbreaks that accounts for uncertainty driven by temporal aggregation of incidence data and case under-reporting. The authors demonstrate their method in application to the Ebola outbreak dataset obtaining more appropriate credible intervals in $R_{t}$ compared with a previously developed approach.

Last, the study by van Osta *et al*. [13] considers an important part of the VV&UQ framework, namely verification of model accuracy. The authors examine a relatively well-known cardiovascular modelling framework, ‘CircAdapt’, which is able to simulate lumped parameter hemodynamics and cardiac dynamics. The authors have previously quantified uncertainties in the framework in both prior and posterior distributions of their parameters [14], yet this work is the first to consider the numerical verification of the model framework. The authors conclude that the implicit multi-step second-order Adams–Moulton numerical method achieved the best verification accuracy in their simulations of left ventricular pressure–volume dynamics.

## Data Availability

This article has no additional data.
